# Disturbances in Pro-Oxidant-Antioxidant Balance after Passive Body Overheating and after Exercise in Elevated Ambient Temperatures in Athletes and Untrained Men

**DOI:** 10.1371/journal.pone.0085320

**Published:** 2014-01-20

**Authors:** Wanda Pilch, Zbigniew Szygula, Anna K. Tyka, Tomasz Palka, Aleksander Tyka, Tomasz Cison, Pawel Pilch, Aneta Teleglow

**Affiliations:** 1 Department of Physiology and Biochemistry, University School of Physical Education, Cracow, Poland; 2 Department of Sports Medicine, University School of Physical Education, Cracow, Poland; 3 Department of Recreation and Biological Regeneration, University School of Physical Education, Cracow, Poland; 4 Rydygier Memorial Hospital, Cracow, Poland; 5 Department of Clinical Rehabilitation, University School of Physical Education, Cracow, Poland; 6 Institute of Physical Education, State Higher Vocational School, NowySącz, Poland; VU University Medical Center, Netherlands

## Abstract

The aim of the study was to investigate pro-oxidant-antioxidant balance in two series of examinations with two types of stressors (exogenous heat and the combined exogenous and endogenous heat) in trained and untrained men. The exogenous stressor was provided by Finnish sauna session, whereas the combined stressor was represented by the exercise in elevated ambient temperature. The men from the two groups performed the physical exercise on a cycle ergometer with the load of 53±2% maximal oxygen uptake at the temperature of 33±1°C and relative humidity of 70% until their rectal temperature rose by 1.2°C. After a month from completion of the exercise test the subjects participated in a sauna bathing session with the temperature of 96±2°C, and relative humidity of 16±5%. 15-minutes heating and 2-minute cool-down in a shower with the temperature of 20°C was repeated until rectal temperature rose by 1.2°C compared to the initial value. During both series of tests rectal temperature was measured at 5-minute intervals. Before both series of tests and after them body mass was measured and blood samples were taken for biochemical tests. Serum total protein, serum concentration of lipid peroxidation products and serum antioxidants were determined. The athletes were characterized by higher level of antioxidant status and lower concentration of lipid peroxidation products. Physical exercise at elevated ambient temperature caused lower changes in oxidative stress indices compared to sauna bathing. Sauna induced a shift in pro-oxidant-antioxidant balance towards oxidation, which was observed less intensively in the athletes compared to the untrained men. This leads to the conclusion that physical exercise increases tolerance to elevated ambient temperature and oxidative stress.

## Introduction

Pro-oxidant-antioxidant balance (PAB) is a state of dynamic balance established under conditions of homoeostasis between free radicals that are created and those consumed (scavenged) [1] This definition concerns cells, body fluids or other human body components. This equilibrium is subjected to permanent dynamic changes caused either by physiological (physical exercise) [2] or pathological factors (illness, presence of xenobiotics or UV radiation) [1]. Physical exercise of great intensity involves muscle damage, which is caused by free radicals that contribute to a more intensive lipid peroxidation [3], [4]. Studies have demonstrated that exercise-induced oxidative damage depends on exercise intensity and duration [5], [6]. One of the elements of body adaptation to regular exercise is elevated concentration of antioxidants, also observed after training sessions [2], [7]. Increased oxidative stress is induced by an increased production of free radicals, reduced number of scavengers and decreased activity of enzymatic systems responsible for their removal, which often coexist with each other [8]. In the active muscle oxidative stress is generated when aerobic metabolism is increased due to the production of reactive oxygen and nitrogen species (RONS) [1].

An important factor which promotes oxidative processes is an increased core body temperature [8]. Physical exercise performed in the a environment leads to considerable dehydration [9] and oxidative stress [10]. Both factors negatively impact the exercise efficiency. Furthermore passive body overheating also shifts the pro-oxidant-antioxidant balance towards oxidation [8].

For this reason the authors emphasized thermal factor when defining research problems. However at this time it is not clear how both factors: passive overheating induced by sauna bathing and physical exercise in elevated ambient temperature modify pro-oxidant-antioxidant balance in human body.

Therefore we conducted this study to investigate modifications in pro-oxidant-antioxidant balance due to only exogenous heat and the combination of exogenous and endogenous heat in athletes and untrained men.

## Materials and Methods

All the men, who took part in the study were fully informed about the protocol and risks associated with the experimental procedure. Being aware of their rights, expressed in the Declaration of Helsinki, all participants have signed the consent to participate in the study. The experiment was approved by the Bioethics Committee for Clinical Research at the Regional Medical Chamber in Cracow, Poland (No. 202KBL/OIL/2011).

### Subjects

Two groups of 10 men took part in the study. They were volunteers, aged 22±0.50 years (Table 1) without any health problems reported in the interview during medical examination and without any contraindications for sauna bathing. The control group (CON) was comprised of untrained men who practised recreational sports. The study group (A) was comprised of professional long distance runners (>5 km) who had similar training experience (6±1.2 years). Mean body mass of the untrained men was 73.4 kg, mean body height was 176.4 cm, mean fat percentage was 10.2% and BMI was 23.6 kg.m^−2^. The athletes were characterized by the following anthropometric indices: mean body height: 179 cm, mean body mass: 67.7 kg, mean fat percentage: 7.1% and BMI of 21.2 kg.m^−2^. The athletes had considerably lower body mass and significantly lower body fat percentage than the untrained men (p<0.05). The athletes exhibited significantly higher (p<0.05) VO_2_max per kg of body mass and significantly higher (p<0.05) respiratory minute volume (V_E_). These indices in athletes were: VO_2_max  = 60.5 ml.kg^−1^.min^−1^ and V_E_  = 118 l.min^−1^ and respectively in the untrained men: VO_2_max  = 44.5 ml.kg^−1^.min^−1^ and V_E_  = 147 l.min^−1^. Two weeks before and during the examinations, the participants did not intake caffeine, alcohol, dietary supplements and vitamins. They also did not take sauna baths on regular basis.

**Table 1:** Participants' age, duration of exercise test, total work, maximal oxygen uptake, respiratory minute volume and duration of sauna bathing session in untrained men (CON) and athletes (A).

Group (Mean±SD)	Age (years)	Duration of exercise test (minutes)	Maximal oxygen uptake [VO_2_max] (ml.kg^−1^.min^−1^)	Respiratory minute volume [V_E_]	Total work [TW]	Duration of sauna bath
				(l.min^−1^)	(kJ)	(minutes)
/CON/	22±0.48	25±9.10	44.5±8.98	118±15.82	167±51.20	34±9.30
/A/	22±0.52	31#±8.90	60.5#±13.51	147#±36.44	269#±13.30	31±9.40

^#^ Significant differences between A and CON at the level of p<0.05.

### Research Protocol

Before the main examinations started, the authors carried out the measurements of the following biometric and structural body indices: body height (BH), body mass (BM), skinfold thickness (suprascapular and triceps skinfold). The results obtained from skinfolds measurements were used for calculation of fat percentage (PF) according to the Slaughter et al. formula [11]. The measurements of body height and mass were used for calculation of body mass index (BMI) according to DuBois and DuBois [12].

The men from both study groups started the primary examinations that comprised of two series.

1. In the first series (1) the effect of physical exercise in elevated ambient temperature 33±1°C on changes in selected physiological indices and changes in the indices of oxidative stress were measured in the men studied.
2. The second series (2) evaluated the effect of passive body overheating during Finnish sauna bathing (temperature of 96±2°C) on changes in the same indices as in the first series of studies.

**Figure 1:**
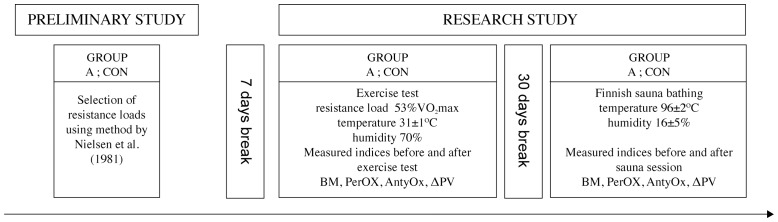
Study design.

Individual resistance loads for all men were adjusted before the first series, using the method proposed by Nielsen et al. [13]. Study design is shown in Figure 1.

The participants (A) and (CON) started the test in the morning (between 8:00 and 11:00 a.m.) after a light breakfast and after previous defecation.

The men had one-week rest after the trials when VO_2_max was determined and suitable resistance load was adjusted, then they performed the test which evaluated the efficiency of exercise-related thermoregulatory mechanisms (first series) [14]. The test procedure was pedalling on a cycle ergometer with individual resistance load determined during initial examinations (53±2%VO_2_max). The exercise test was performed on a cycle ergometer ER 900 D – 72475 BIT 2 (Jaeger, Germany). The exercise was carried out in the climatic chamber at the temperature of 33±1°C and relative humidity of 70% until rectal temperature rose by 1.2°C. During the exercise men were wearing shorts and athletic footwear.

The second series was carried out after a month. It consisted in passive heating of the subjects' bodies in Finnish sauna with the temperature 96°±2°C at face level and the relative humidity of 16±5%. Men stayed for 15 minutes in a hot sauna chamber in a reclining position. The sauna session was finished with a 2-minute cool-down in the shower with constant water temperature of 20°C. These activities (15-minutes heating and 2-minute cool-down) were repeated until subjects' rectal temperature (Tre) rose by 1.2°C compared to the initial value.

### Measurements

The following indices were measured during the first and the second series of the examinations: body mass (BM) before exercise, before sauna bathing, after the exercise and after sauna bathing.The subjects were weighed naked using Sartorius electronic scales (F 1505 – DZA, Germany).

Rectal temperature (Tre) was monitored in both series at 5-minute intervals using CTD85M electric thermometer (Ellab, Denmark) with the accuracy of 0.1°C.

### Blood tests and biochemical analyses

Before each series of the examinations (1 and 2) and 3 minutes after them, the heparynised blood samples (15 ml) were taken (always in the same sitting position) to test tubes from the cubital vein and then centrifuged for 10 minutes in a K-24 centrifuge (Janetzki, Germany). The serum obtained with this method was stored in a refrigerator at the temperature of −80°C. After completion of all the samples in the serum which was free of haemolysis traces, the following biochemical indices were determined: total protein using biuret method by means of Hitachi 917 Modular P analyser, concentration of lipid peroxidation products (PerOx) and plasma antioxidant concentration (AntyOx)with ImmunDiagnostik tests using ELISA (method sensitivity 7 µmol.l^−1^).

Calculations of the changes in plasma volume (ΔPV) were made based on the concentration of total protein determined before and after both series of the examinations using the Harrison's formula [15]:
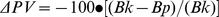
where: Bp – initial protein determined before the examination, Bk – final protein determined after the examination.

Due to dynamic changes in plasma volume that occur during physical exercise in the climatic chamber and during sauna bathing, the oxidative stress indices, measured after exercise and after sauna exposure to heat, were corrected for plasma volume changes [16] using Kraemer and Brown's formula [17].
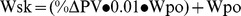
where: W _sk_- corrected value, W_po_ – value after the exercise or after sauna bathing.

### Statistical Analysis

In order to check normality of data distribution, authors used Shapiro-Wilk test. Student's t test was used for all variables (both dependent and independent) as the results obtained normal distribution. Statistical significance between the means was set at p<0.05. The relationships between the individual indices were determined using Pearson's linear correlation coefficients with the statistical significance set at p<0.05.

## Results

Mean duration of sauna bathing, until reaching the increase in rectal temperature of 1.2°C, was similar in both groups (A and CON) (Table 1). Mean duration of exercise at intensity of 53%VO_2_max in elevated ambient temperature (33±1°C) was significantly longer (p<0.05) in athletes compared to the untrained. The amount of work done during the exercise test was significantly higher (p<0.05) in athletes compared to the untrained (Table 1).

A statistically significant (p<0.05) body mass loss, due to dehydration caused by sweating during sauna bathing,was observed in both groups (Table 2). Statistically significantly higher body mass loss expressed as percentage of the initial body mass was observed in the athletes both after the exercise test and after sauna bathing (p<0.05). These changes amounted on average in athletes to (−)1.2% after performing the exercise test and (−)1.43% after sauna bathing. Lower percentage body mass loss was recorded in the untrained men. They were respectively (−)0.72% after the exercise test and (−)0.9% after overheating in sauna.

**Table 2:** Changes in body mass (BM) after performing the exercise test and after sauna bathing in untrained men (CON) and athletes (A).

Variable (Mean±SD)	Group	Before	After	Δ
	Exercise test/CON/	72.51±8.56	71.99*±8.46	−0.52±0.15
BM (kg)	Exercise test/A/	67.78±5.35	66.98*±5.36	−0.8^#∧^±0.16
	Sauna/CON/	72.99±9.34	72.44*±9.30	−0.55±0.40
	Sauna/A/	68.22±5.46	67.27*±5.44	−0.95#±0.28

Statistically significant differences at p<0.05 compared to the levels before sauna bathing and before the exercise test.

^#^ Significant differences at the level of p<0.05 between A and CON.

Statistical differences at the level of p<0.05 between exercise test and sauna bathing in the group A.

Both physical exercise in elevated temperature and sauna bathing caused considerably higher decline in plasma volume in the athletes compared to the untrained men (p<0.05) (Table 3).

**Table 3:** Changes in plasma volume (%ΔPV) in the groups CON and A after exercise test and after sauna bathing.

Variable (Mean±SD)	Group	Δ
	Exercise test/CON/	−8.0±1.70
%ΔPV	Exercise test/A/	−10.5#±3.20
	Sauna/CON/	−7.0±3.30
	Sauna/A/	−10.2#±3.30

^#^ Statistically significant at p<0.05 A compared to CON.

The athletes' blood before sauna bathing exhibited higher concentration of antioxidant status and twice lower concentrations of lipid peroxidation products compared with the untrained group at p<0.05 (see Table 4). Exposure to high temperature during sauna bathing reduced antioxidant status in the untrained subject by almost three times (p<0.05) and intensified the process of peroxidation by 38.4% (p<0.05). The changes caused by sauna bathing in athletes were considerably less intensive and concerned the reduction in plasma antioxidant status by 20.8% (p<0.05) and increase in concentration of peroxidation products by 56.2% (p<0.05). Analysis of the indices revealed a negative relationship between reduction in the concentration of plasma antioxidant status in the untrained men and duration of the exercise in elevated ambient temperature (r = −0.55; p<0.05).

**Table 4:** Changes in concentration of lipid peroxidation products and antioxidant status in blood plasma in CON and A subjects during sauna bathing.

Variable (Mean±SD)	Group	Before	After	Δ
Antioxidant status	Sauna/CON/	200.57±44.57	69.14*±23.57	−131.40±49.64
(µmol.l^−1^)	Sauna/A/	250.25#±44.58	198.25* ^#^±23.57	−52.00#±36.14
Peroxidation products	Sauna/CON/	139.62±52.53	193.25*±66.28	53.62±20.66
(µmol.l^−1^)	Sauna/A/	65.28#±20.16	102.00* ^#^±26.96	36.70#±33.80

Statistically significant at p<0.05 compared to the levels before sauna bathing.

^#^ Statistically significant at p<0.05 compared to the group CON.

Physical exercise performed in elevated ambient temperature caused reduction in the antioxidant status by 24.3% (p<0.05) and more intensive (p<0.05) lipid peroxidation in the group of the untrained (Table 5). The changes observed in the group of athletes were: reduction in antioxidant status by 21.3% (p<0.05) and almost twice higher level of lipid peroxidation products (p<0.05). A negative relationship was also observed between reduction in the level of blood antioxidants in the trained men and the work done by them at elevated ambient temperature (r = −0.77; p<0.05).

**Table 5:** Changes in concentration of lipid peroxidation products and antioxidant status in blood plasma in CON and A subjects during exercise test.

Variable (Mean±SD)	Group	Before	After	Δ
Antioxidant status	Exercise test/CON/	220.42±49.50	166.85*±53.80	−53.57±50.10
(µmol.l^−1^)	Exercise test/A/	242.40#±37.10	190.55* ^#^±37.80	−51.85±37.40
Peroxidation	Exercise test/CON/	113.17±42.57	183.00*±69.81	69.83±51.80
products (µmol.l^−1^)	Exercise test/A/	67.20#±27.43	127.00* ^#^±71.36	59.80±49.10

Statistically significant at p<0.05 compared to the levels before exercise.

^#^ Statistically significant at p<0.05 A compared to CON.

## Discussion

According to Kubica et al. [18] interindividual variability concerning the rate of core temperature rise is associated with exercise thermoregulation which determine ability to work in hot environments. Time to increase rectal temperature by 1.2°C is an indicator of exercise thermoregulatory mechanisms' efficiency [14]. In our study the time to increase rectal temperature by 1.2°C during exercise in the climatic chamber was longer in athletes than in untrained men. Total work performed by the athletes was significantly higher compared to untrained men which suggests greater efficiency of exercise thermoregulatory mechanisms. This indicates that running training regimes carried out for many years, in thermoneutral ambient conditions improved exercise thermoregulatory mechanisms. It has been reported that trained runners are characterized by high heat tolerance [19], which is further increased due to training [20], [21], [22].

Dehydration which is expressed by the decline in body mass observed in athletes, both after exercise in elevated ambient temperature and after sauna bathing, was significantly higher (p<0.05) than the dehydration observed in the untrained in both series of examinations. This suggests better thermoregulation in the first group. It is supported by an increased body water percentage, greater sweat rate and more advanced angiogenesis of large muscle groups. Intensive sweating during both examinations' series caused a greater decline in plasma volume (p<0.05) in athletes. This suggests their better adaptation to exercise in hot environment and more effective heat elimination. Higher body mass loss and consequently more intensive dehydration observed after exercise in an elevated ambient temperature and after sauna bathing in athletes could have been connected with higher body water percentage and less fat tissue. Body fat percentage in the untrained men was higher while body mass loss caused by sweating was lower. It should be emphasized that endurance training causes an increase in body plasma volume [23].

During intensive exercise, mainly endurance type, the oxygen intake is increased by 10–20 times, while its consumption in tissues increases by 100–200 times compared to the conditions at rest [24]. The increase in the amount of reactive oxygen species (ROS) and reactive nitrogen species (RNS) in human body is caused by intensified intercellular metabolism rate [1], [7], [24]. Generation of ROS and RNS (i.e. RONS) promotes lipid peroxidation process, denaturation of proteins, inactivation of enzymes, modification of DNA and RNA, that create huge threat to proper functioning of cells causing the disturbance in their structure and function in human body [25]. An increased auto-oxidation of oxyhaemoglobin to methemoglobin during physical exercise is also conductive to ROS formation and leads to the impairment of the anti-oxidative blood system [26]. Haem and iron released during denaturation of haemoglobin are the factors activating free radical processes which disturb pro-oxidant-antioxidant balance during physical exercise [27], [28].

According to the examinations carried out by Vincent et al. [29], [30] it is sufficient to perform endurance training for five days in order to induce considerable improvement in both oxidative and anti-oxidative potential of skeletal muscles. Physical exercise might change the activity of antioxidant enzymes and it was demonstrated that these changes differ in tissues and organs. It was found that physical exercise has considerable effect on the activity of anti-oxidative enzymes in skeletal muscles and that these enzymes are more active in the muscles dominated by aerobic metabolism [31], [32]. The adaptive increase in anti-oxidative enzyme activity induced by training shows that body defence against RONS is more efficient in the people with better training status, which is suggested by correlations between activity of these enzymes in the muscles and VO_2_max [33]. Well-trained athletes with higher aerobic threshold exhibit higher blood antioxidant capacity [34]. This suggests that endurance training might be an important element to sustain and intensify the athlete's potential and to give the protection against oxidative stress [32], [35]. In our study similar to other authors [33], [34], [36], [37], it was observed that before exercise and sauna bath, the plasma antioxidant status in long distance runners was higher than in the untrained subjects.

Hyperthermia might intensify oxidative stress through decoupling of mitochondrial respiratory chain or through inhibiting anti-oxidative defence mechanisms. A considerable increase in concentration of lipid peroxides was found in young men whose core temperature elevated to 39.5°C during performing 50%VO_2_max treadmill exercise in a hot environment compared to the untrained men who performed the same exercise at a thermoneutral temperature with the same oxygen uptake. Therefore, hyperthermia elevates oxidative stress and selectively affects specific oxidative markers, regardless of oxygen intake [38].

The exercise performed at elevated ambient temperature increases oxidative stress that accompanies physical work [39]. Furthermore, dehydration caused by exercise intensifies oxidative stress [40]. This was also confirmed in our study. Laitano et al.[41] demonstrated that submaximal exercise performed in a hot environment by young men increased oxidative stress, which was measured by changes in the activity of superoxide dismutase (SOD), glutathione (GSH), and glutathione disulfide (GSSG). The most noticeable changes in activity of these enzymes occurred in men, whose dehydration amounted to 3.5% of body mass loss compared to the group where re-hydration procedures were used.

Stressors activate mechanisms of defence against stress which first involve the following molecules: α-tocopherol, ascorbic acid, glutathione, Q coenzyme, uric acid, superoxide dismutase, glutathione peroxidase and other compounds. They can also involve heat-shock proteins (HSP) released, among other things, from the liver as a response to stress. Due to the increased HSP levels induced by oxidative stress, these proteins are also reported to have an antioxidant effect [42], [43].

Increase in body temperature during sauna bathing leads to hyperventilation and increased oxygen intake [44]. Elevated blood oxygen concentration leads to elevated production of reactive oxygen species [45], [46], leading to disturbed pro-oxidant-antioxidant balance, which was observed in a study by Zinchuk and Zhadzko [46], who exposed athletes to a single and repeated sauna bathing sessions. These authors observed (after hyperthermia) an increase in nitrogen oxide synthesis in the subjects' higher concentration of nitrides/nitrites and elevated concentrations of lipid peroxidation products [46]. In our study, we also found a disturbed pro-oxidant-antioxidant balance after sauna bathing through increased concentration of lipid peroxidation products while reduced plasma antioxidant concentration in both athletes and untrained men.

The exercise training causes greater adaptation to oxidative stress due to the stimulation (through ROS) of transcription factors NF-κB and AP-1 which control expression of over 100 genes, including antioxidant enzymes. Fehrenbach and Northoff [42] found that ROS are the molecules that also control activity of the HSF transcription factor which is responsible for the expression of HSP70, HSP72 and HSP90 proteins. This means that the stressors generated during exercise stimulate changes which lead to strengthening of defence against oxidative and thermal stress [42], [43], [47]. This pattern was noticeable in our experiment when comparing the antioxidant status and concentration of peroxidation products before and after sauna bathing. The athletes were characterized by higher level of antioxidant status and lower concentration of lipid peroxidation products. It was demonstrated for the first time that physical exercise at elevated temperature caused lower changes in the oxidative stress indices compared to passive overheating in sauna, with the same increase in core body temperature.

## Conclusions

The study demonstrated that:

1. Both passive and active overheating of human body led to disturbed pro-oxidant-antioxidant balance.
2. The athletes had initially higher antioxidant status level and lower plasma peroxidation products level compared to the untrained men, and, in both series, the disturbances of their pro-oxidant-antioxidant balance remained lower.
3. Oxidative stress differed depending on a thermal stressor. Physical exercise at elevated ambient temperature caused lower changes in the oxidative stress indices compared to sauna bathing.
